# The United States Veterinary Medical Association

**Published:** 1894-09

**Authors:** 


### EDITORIAL.

---

### THE UNITED STATES VETERINARY MEDICAL ASSOCIATION.

Another annual meeting is close upon us, and the result of our coming together will be a matter of history in the literature of our profession. Whether we take another step forward in the phenomenal progress which has crowned our efforts during the past few years, or whether we have rested upon our oars as the result of an unprecedented activity at the international congress of last year, will soon be known. That we will not show the lack of enthusiasm which so often marks the meetings of all societies following one especially active and important, is most sincerely to be hoped.

In our youth especially we cannot afford to lag in the least, and each individual member should and must feel that he is responsible to a certain extent for the continued prosperity and influence of the association. The success of our association affects the personal interests of every honorable and honest member of our profession. That much good committee work has been performed during the present year, we are in a position to state absolutely. Matters which concern the welfare of the profession have received very careful consideration, and the reports of these committees of themselves will be of sufficient importance to demonstrate the necessity of such organized work as can only be performed by such an association as ours Doubtless every member of the association has had his income lessened by the financial depression through which the country is passing, but the expense attendant upon a visit to Philadelphia the third week in September will more than repay one. Interesting papers will be presented to the members present, and it is to be hoped that a truly national meeting may be held. The East has always turned out in force when the meetings have been held in the West, and now let our professional brothers from all over the country return the compliment by coming East this year.

